# Detecting cell-secreted growth factors in microfluidic devices using bead-based biosensors

**DOI:** 10.1038/micronano.2017.25

**Published:** 2017-07-03

**Authors:** Kyung Jin Son, Pantea Gheibi, Gulnaz Stybayeva, Ali Rahimian, Alexander Revzin

**Affiliations:** 1 Department of Biomedical Engineering, University of California, Davis, California 95616, USA; 2 Department of Physiology and Biomedical Engineering, Mayo Clinic, Rochester, Minnesota 55905, USA

**Keywords:** biosensors, growth factors, microbead, microchamber, primary hepatocytes

## Abstract

Microfluidic systems provide an interesting alternative to standard macroscale cell cultures due to the decrease in the number of cells and reagents as well as the improved physiology of cells confined to small volumes. However, the tools available for cell-secreted molecules inside microfluidic devices remain limited. In this paper, we describe an integrated microsystem composed of a microfluidic device and a fluorescent microbead-based assay for the detection of the hepatocyte growth factor (HGF) and the transforming growth factor (TGF)-β1 secreted by primary hepatocytes. This microfluidic system is designed to separate a cell culture chamber from sensing chambers using a permeable hydrogel barrier. Cell-secreted HGF and TGF-β1 diffuse through the hydrogel barrier into adjacent sensing channels and are detected using fluorescent microbead-based sensors. The specificity of sensing microbeads is defined by the choice of antibodies; therefore, our microfluidic culture system and sensing microbeads may be applied to a variety of cells and cell-secreted factors.

## Introduction

Microfluidic devices for cell cultivation have garnered considerable interest because they are more economical in terms of cells and reagents, allow precise control over the composition and rates of flow streams, and may be automated through the use of valves/actuators^1^. In addition to the advantages listed above, several reports have indicated that endogenous signals may play a prominent role in microfluidic cell cultures operating under low-flow conditions^2,3,4,5^. For example, work in our laboratory demonstrated that embryonic stem cells, cancer cells, and primary hepatocytes exhibited markedly different phenotypes inside microfluidic devices operated under diffusion-dominated conditions compared with those inside standard multiwell plates^6,7,8^. This phenotype enhancement was due, at least partly, to the rapid accumulation of endogenous signals inside microfluidic channels, underscoring the need for biosensors that locally monitor the levels of these signals. In our opinion, these biosensors should be miniature, allow for the local monitoring of secreted factors, and be compatible with long-term cell cultures. The latter point is both important and challenging to implement, particularly with affinity biosensors, because of the rapid saturation of the binding site by the target molecules. Typically, sensing cell secretions in microfabricated cultures involves immobilizing biorecognition molecules such as antibodies (Abs) or aptamers in close proximity to cells^9,10,11,12,13^. These approaches are powerful but have proven challenging to implement for long-term cell cultures due to the rapid saturation of immobilized biorecognition molecules. The lifetime of such biosensors may be extended by incorporating valving or reconfigurable microfluidic channels, but this is done at the expense of simplicity^14,15^. Another strategy has been specifically developed to work with the “microengraving” method^16^, whereby cells reside in polydimethylsiloxane (PDMS) microwells sealed by an Ab-modified glass coverslip. The coverslip retains the cell-secreted molecules and can be exchanged at different time points to alleviate the problem of saturation^17,18, 19^. Although very useful, this strategy was designed specifically for the “microengraving” method and is not broadly applicable to sensing inside microfluidic devices. We reasoned that instead of stationary biosensors (for example, immobilized aptamers or Abs), it may be fruitful to pursue mobile, bead-based biosensors that can be introduced into a microfluidic channel for a prescribed period of time and then replaced by a fresh batch of sensing microbeads.

Microbeads are widely used for sensing in the context of microfluidic channels or microcapsules^20,21^. Recently, we described the incorporation of microbeads into PDMS microcompartments alongside single cells, and demonstrated the detection of cell-secreted proteins and exosomes^22^. The sensing assay consisted of Ab-modified microbeads and free-floating fluorescent Abs. The binding of cell-secreted molecules to microbeads caused free-floating Abs to assemble, forming fluorescent sandwich complexes on microbeads. The fluorescence increased dynamically over time and was correlated with the concentration of cell-secreted factors. A similar microbead-based assay was recently reported for monitoring the cytokine production from single immune cells^23^.

**Figure 1:**
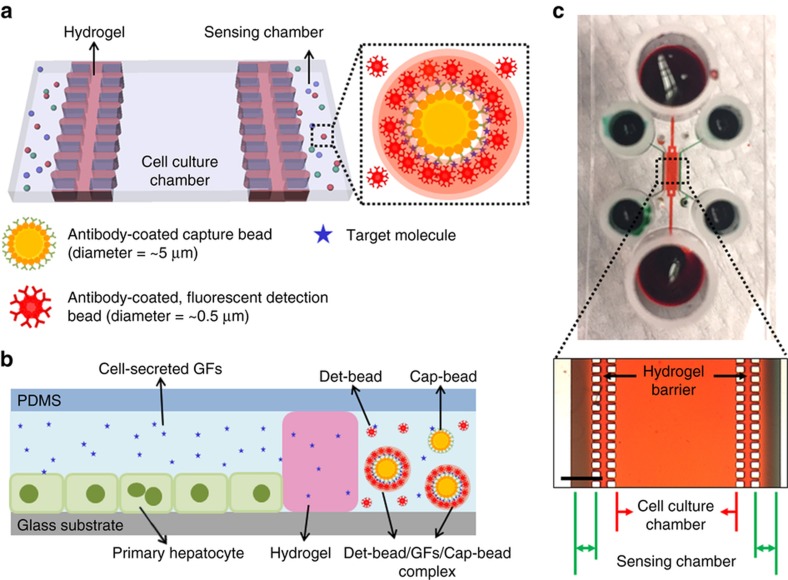
A microsystem for the cultivation of hepatocytes and for the detection of secreted growth factors. (**a** and **b**) Schematic of a microfluidic device containing a hydrogel barrier between the cell culture chamber (middle) and the sensing chambers (side). Growth factors produced by hepatocytes diffuse through the hydrogel barrier and cause the sensing beads to aggregate. The appearance of fluorescence on the capture beads is used as a readout of binding events. (**c**) A photograph and microscopic image of a microfluidic device. Red and green food dyes were infused into a cell culture chamber and sensing chambers, respectively. Scale bar=500 μm. GF, growth factor.

In this paper, we used sensing microbeads in conjunction with microfluidic cultures of primary hepatocytes. The need for biosensors was motivated by our recent observation that hepatocytes in microfluidic channels upregulate the production of the HGF, while downregulating the production of the TGF-β1 Ref.(7). Enzyme-linked immunosorbent assays (ELISAs) used previously by us were not well suited for measuring the local concentrations of these growth factors (GFs) at the site of cells. To remedy this, we designed a microfluidic device with parallel microchambers for the cultivation of cells and the detection of secreted GFs (Figure 1). The chambers were separated by a hydrogel barrier that allowed secreted factors to diffuse unimpededly, while preventing cells from crossing over into sensing channels. Ab-modified microbeads were infused into sensing microchannels at desired time points during the culture and were used to monitor the local concentrations and secretion rates of the HGF and TGF-β1 over the course of 7 days. The cell culture and sensing microsystem described here address the need for the local and long-term detection of cell-secreted factors inside microfluidic channels. This system may be easily adapted to accommodate a variety of cell types and cell-secreted signals.

## Materials and methods

### Materials

Streptavidin-coated polystyrene particles (diameter=5.0–5.9 μm) and streptavidin-coated fluorescent polystyrene particles (diameter=0.4–0.6 μm; Nile Red and Yellow) were purchased from Spherotech (Lake Forest, IL, USA). Biotinylated goat anti-HGF Abs (anti-HGF Ab-biotin) and biotinylated chicken anti-TGF-β1 Abs (anti-TGF-β1 Ab-biotin) were purchased from R&D Systems (Minneapolis, MN, USA). Poly(ethylene glycol) diacrylate (PEGDA, molecular weight (MW) 700 Da) and poly(ethylene glycol) (PEG, MW 2000 Da and 20 000 Da) were purchased from Sigma (St Louis, MO, USA). 1-[4-(2-Hydroxyethoxy)phenyl]-2-hydroxy-2-methyl-1-propan-1-one (Irgacure 2959) was purchased from Ciba Specialty Chemicals (Basel, Switzerland). Phosphate-buffered saline (PBS) was purchased from TEKnova (Hollister, CA, USA). Glass slides (75×25 mm^2^) and cover glasses (24×30×0.13 mm) were purchased from Thermo Fisher Scientific (Pittsburgh, PA, USA). Cell culture reagents and supplies were purchased from Gibco and Invitrogen both of which are subsidiaries of ThermoFisher Scientific. All other chemicals were purchased from Sigma-Aldrich Chemicals (Milwaukee, WI, USA). COMSOL Multiphysics (COMSOL, Inc., Burlington, MA, USA) was used for numerical simulation to determine the concentrations of cell-secreted GFs inside microfluidic devices. ImageJ software with a particle-tracking plugin (National Institutes of Health, Bethesda, MD, USA) was used for image processing of the fluorescence images of the sensing beads.

A custom-built cell culture chamber was placed on a Nikon Eclipse TI fluorescence microscope (Nikon Instruments Inc., Melville, NY, USA) to maintain the physiological conditions (37 °C, 5% CO_2_) during sensing sessions.

Primary hepatocytes were isolated from adult female Lewis rats weighing 125–200 g (Charles River Laboratories, Boston, MA, USA) using a two-step collagenase perfusion procedure^24^. Primary hepatocytes were maintained in Dulbecco’s modified Eagle medium (DMEM; Gibco, 11995), supplemented with 10% (v/v) fetal bovine serum (Invitrogen), 200 U mL^−1^, penicillin (Invitrogen), 200 mg mL^−1^ streptomycin (Invitrogen), 7.5 mg mL^−1^ hydrocortisone sodium succinate (Sigma), 20 ng mL^−1^ epidermal GFs (Invitrogen), 7 ng mL^−1^ glucagon (Sigma), and 0.5 U mL^−1^ human insulin (Novolin N) at 37 °C in a humidified 5% CO_2_ atmosphere. All experiments were performed under the National Institutes of Health (NIH) guidelines for the ethical care and use of laboratory animals, and the experimental protocol was approved by the Institutional Animal Care and Use Committee of the University of California, Davis.

### Ab immobilization on microbeads

A set of microbeads consisted of capture microbeads (non-fluorescent; diameter=5.0–5.9 μm) and detection microbeads (fluorescent Nile Red for the HGF and Yellow for the TGF-β1; diameter=0.4–0.6 μm). Both types of beads were streptavidin-coated by the manufacturer and were incubated with biotinylated anti-GF Abs as follows. First, microbeads were washed with PBS three times using a centrifugation/washing protocol (12 000 r.c.f. for 3 min, Centrifuge 5424, Eppendorf, Hamburg, Germany). Capture microbeads (~1.4×10^6^ beads or 0.1 mg) were then incubated overnight at 4 °C with 4 μg of biotinylated Abs in 50 μL of PBS solution containing 1% bovine serum albumin (BSA). Detection microbeads were prepared in a similar manner. Five microgram of streptavidin-coated fluorescent microbeads (~2.0×10^8^ beads) was incubated overnight at 4 °C with 2 μg of biotinylated Abs in 50 μL of PBS containing 1% BSA. Both capture/detection microbeads functionalized with Abs were washed with PBS three times, followed by blocking with 2% BSA for 30 min at room temperature. Ab microbeads were stored for up to 2 weeks at 4 °C.

### Incorporating hydrogel barriers into microfluidic devices

A microfluidic device contained a cell culture chamber (5 mm (*L*)×1.5 mm (*W*)×60 μm (*H*)) flanked by sensing chambers (5 mm (*L*)×250 μm (*W*)×60 μm (*H*)) on both sides (Supplementary Figure S1A). The microfluidic devices were fabricated using the standard soft lithographic methods. First, a master wafer was prepared by patterning SU-8 2050 (Microchem Corp., Woburn, MA, USA). Subsequently, liquid PDMS (Sylgard 184, Dow Corning, Midland, MI, USA) was mixed with a curing agent at a 10:1 weight ratio, poured onto a silicon master, degassed under a vacuum for 30 min to remove air bubbles, and cured at 70 °C for 1 h. Silicone slabs with imprinted microchannels were cut out from the wafer. A biopsy punch (diameter=5 mm) was then used to make inlet and outlet ports for the sensing and cell culture chambers. A smaller biopsy punch (diameter=1 mm) was used to make the inlet and outlet ports needed to infuse the PEG prepolymer and to form a hydrogel barrier. Cloning cylinders (diameter=6 mm for sensing chambers and 10 mm for the cell culture chamber; Fisher Scientific) were attached using the PDMS prepolymer solution to serve as reservoirs, and devices were further baked at 70 °C for 20 min. For irreversible bonding, both the PDMS slabs and the glass coverslips were treated with oxygen plasma for 3 min at 300 W and then brought into contact. The microfluidic devices were placed at 70 °C overnight to restore their surface hydrophobicity.

We prepared a series of PEG prepolymer solutions to test the diffusivities of the resultant hydrogels. The variants tested included 5% (v/v) PEGDA with 5% (w/v) 20 k PEG, 5% (v/v) PEGDA with 10% (w/v) 2 k PEG, or 10% (w/v) 20 k PEG, and 5% (v/v) PEGDA with 20% (w/v) 2 k PEG or 20% (w/v) 20 k PEG. All PEG prepolymer solutions contained 1% (w/v) Irgacure 2959. A prepolymer solution was manually infused into specially designed channels that were located between the cell and sensing chambers. Instead of contiguous walls, these channels were formed by two columns of PDMS posts (height×length×width of 75 μm×100 μm×60 μm, respectively, and vertical edge-to-edge distance of 37 μm) (Supplementary Figure S1B). The width of these channels was 80 μm. Empirically, we determined this to be the minimal width needed to contain the prepolymer solution and to prevent it from spilling into the adjacent cell and sensing chambers. Liquid PEG propolymer inside the channels was polymerized by allowing 30 s of exposure to 365 nm ultraviolet (UV) light (146 mW cm^−2^; OmniCure Series 1000, Lumen Dynamics Group, Mississauga, Ontario, Canada). Subsequently, all chambers of the microfluidic device were filled with PBS and stored at 4 °C for 2–5 days to remove any unpolymerized PEG and to make the channel surfaces hydrophilic.

### Numerical simulations performed to determine the concentration gradients of cell-secreted GFs inside microfluidic devices

Numerical simulations were performed to estimate the concentrations of cell-secreted HGF and TGF-β1 in the cell culture chamber (local concentration) and in the whole microfluidic device (global concentration) using the COMSOL Multiphysics 4.3 software (COMSOL Inc., Los Angeles, CA, USA). We assumed that (1) cells secrete GFs at a constant rate, and that (2) there is back and forth movement of the flow due to differences in surface tension between the inlets and outlets of media reservoirs. For simplification, we further assumed that this back and forth flow movement followed a waveform function with an angular frequency of 1.45×10^−4^, phase of π/2, and amplitude of 0.5 μm s^−1^. The cell-secreted GF levels in a low-flow microfluidic device can be estimated by solving the following equation:

$$\frac{\partial c_{i}}{\partial t}=\nabla \cdot \left(D_{i}\nabla c_{i}\right)-u\cdot \nabla c_{i}+R_{i}$$

where *c*_*i*_ is the cell-secreted GF concentration (pM), *D*_*i*_ is the diffusivity of the cell-secreted GF (cm^2^ s^−1^) in the medium and in the hydrogels, and *u* is the estimated flow velocity (μm s^−1^). *R*_*i*_ denotes the endogenous GFs secreted by cells, which can be described as follows:

$$R_{i}=\sigma_{\sec}\cdot \rho_{\mathrm{cell}}$$

where *σ*_sec_ is the GF secretion rate (mol s^−1^ per cell) and *ρ*_cell_ is the average cell density, which was experimentally determined. All parameters used for the simulations are as follows:


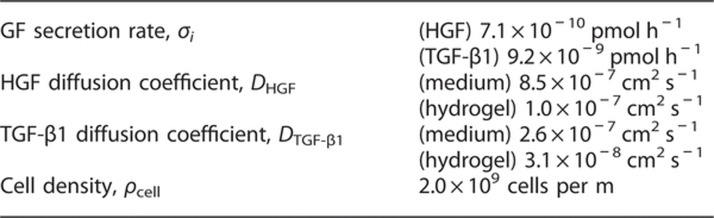


### Calibration of microbead biosensors

Prior to the cell experiments, calibration curves for the HGF and TGF-β1 were generated by challenging microbeads with different concentrations of recombinant GFs. To mimic a cell secretion experiment, a solution of recombinant GFs was injected into a cell culture chamber, whereas sensing beads were infused into a sensing chamber. A typical calibration experiment proceeded as follows. First, 100 μL of media containing the desired concentration of GF was dispensed into the inlet of the cell culture chamber. After infusion, the amount of media in both reservoirs was balanced by adding 100 μL of media containing the recombinant GFs into the outlet (the other reservoir). Simultaneously, 50 μL of media containing 7.0×10^5^ capture beads and 1.0×10^8^ detection beads was injected into the sensing chambers. The change in fluorescence of the capture microbeads was monitored for 90 min.

### Detecting HGF and TGF-β1 secreted by hepatocytes in microfluidic devices

To detect the GF secretion of the primary hepatocytes, the microfluidic devices were sterilized under UV exposure for 30 min and coated with 0.2 mg mL^−1^ of collagen type I (BD Biosciences, San Jose, CA) for 1 h at 37 °C, followed by washing with PBS and cell culture media. After aspirating the media from all reservoirs of the device, 50 μL of the media containing the primary hepatocytes (~10^6^ cells per mL^−1^) was inserted into the inlet and allowed to flow into the channel, driven by the difference in liquid head between the inlet and outlet ports. Cells residing in the inlet and the outlet were removed, and 250 μL of media was added to each reservoir. A measure of 50 μL of media was additionally added to the sensing chambers. The device was kept in a tissue culture incubator (5% CO_2_ at 37 °C) overnight to allow the cells to attach to the floor of the cell culture chamber, followed by the washing of unbound hepatocytes with fresh media. The devices seeded with hepatocytes were kept inside a tissue culture incubator for 7 days, with changing of the media occurring daily.

Sensing sessions were carried out on days 1, 4, and 7. First, the media in the sensing chambers were replaced by 50 μL of media containing capture beads (7.0×10^5^ beads for each GF) and detection beads (1.0×10^8^ beads for each GF). We presumed that no residual GFs existed in the sensing chamber after media replacement; therefore, only the GFs that diffused from the cell culture chamber to the sensing chamber during the sensing sessions were detected. The microfluidic device was placed inside the microscope-mounted environmental chamber described above, and the fluorescence signal was monitored over the course of 90 min. After the sensing sessions were conducted, the media in the cell culture chamber were replaced.

## Results and discussion

### Design of microfluidic devices for cell culture and GF detection

**Figure 2:**
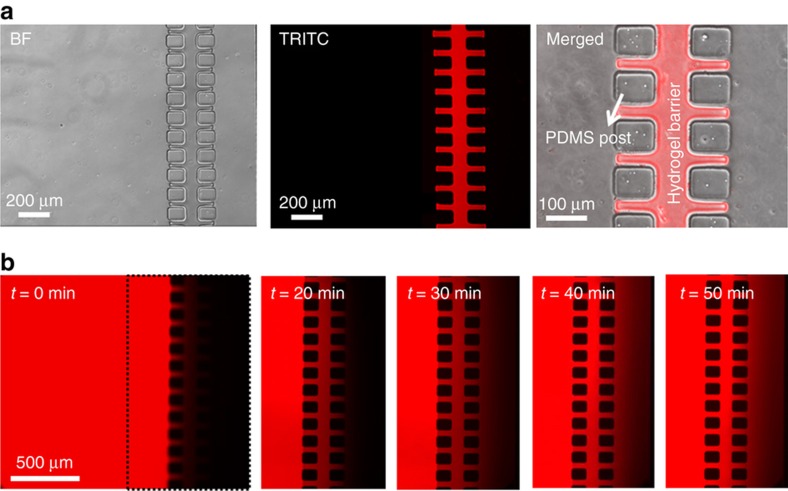
Characterization of the diffusion properties of hydrogel barriers built into microfluidic chambers. (**a**) Bright-field, fluorescence, and merged images showing the fluorescently labeled 5% PEGDA/20% 20 k PEG hydrogel barrier inside a microfluidic device. (**b**) Characterization of the diffusion of 75 kDa TRITC-dextran through a 5% PEGDA/20% 20 k PEG hydrogel barrier inside a microfluidic device. A solution of TRITC-dextran (2.5 mg mL^−1^ in PBS) flowed into the cell culture chamber (left) and diffused through the hydrogel barrier toward the sensing chamber (right). PBS, phosphate-buffered saline; PEG, poly(ethylene glycol); PEGD, Poly(ethylene glycol) diacrylate; PDMS, polydimethylsiloxane.

This paper describes the development of a microfluidic device and sensing microbeads for the cultivation of cells and the on-chip detection of secreted GFs. A key feature of this device (shown in Figure 2 and Supplementary Figure S1A) was a thin hydrogel barrier that separated the cell culture chambers from the sensing chambers. This design allowed the sensing microbeads to be in close proximity with the cells but not in the same compartment with the cells. Our initial efforts were to design a hydrogel barrier to permit the diffusion of secreted GFs. First, we used the COMSOL Multiphysics program to model the diffusion of the HGF through hydrogel barriers of different diffusion coefficients (*D*_g_). Supplementary Figure S1B shows the HGF concentration profiles in the sensing chamber as a function of time. The rate of HGF secretion was taken to be 6.5×10^−5^ pg h^−1^ per cell based on our previous experiments, with the assumption that 1.5×10^4^ hepatocytes were present in the cell culture chamber^25^. The diffusion coefficient of the HGF in the solution (*D*_0_) was calculated to be 7.6×10^−7^ cm^2^ s^−1^ using the Stokes–Einstein equation^26^. We then plotted the concentration of the HGF over time in the sensing chamber for different gel diffusion coefficients (*D*_g_), which were considered a fraction of the HGF solution diffusion coefficient (*D*_g_/*D*_0_). This modeling revealed that *D*_g_ needs to be one-tenth of *D*_0_ to allow a considerable amount of the HGF (~20% at *t*=90 min) to cross the hydrogel barrier. Therefore, subsequent optimization experiments focused on creating hydrogel barriers with a *D*_g_ value of 0.8×10^−7^ cm^2^ s^−1^. We should also note that the thickness of the hydrogel barrier is an important parameter governing the permeation of GFs. The minimal width of the hydrogel barrier was limited by our fabrication capabilities to 80 μm.

It is of note that the diffusion coefficient of analytes strongly depends on the mesh size of the hydrogel network^27,28^. PEGDA hydrogels prepared, without crosslinkers, from oligomers ranging in MW from 575 Da to 20 kDa are expected to have mesh sizes of 0.1–10 nm Refs.(29,30,31).The hydrodynamic radius (*R*_H_) of the HGF is reported to be ~4 nm Ref.(32). We surmised that diffusion may be challenging in a scenario where the protein molecules and pores are of the same length scale^33^. Therefore, we followed previously published reports to incorporate porogens into the gel to further enhance its porosity and diffusivity^34,35,36,37,38^. PEG without a crosslinkable functional group was chosen as a porogen. Different formulations composed of chemically active and inactive PEG molecules were tested. These formulations included 5% (v/v) PEGDA (MW 700 Da) with 5% (w/v) 2 k PEG, 5% (w/v) 20 k PEG, 10% (w/v) 2 k PEG, 10% (w/v) 20 k PEG, 20% (w/v) 2 k PEG, or 20% (w/v) 20 k PEG. Prepolymers of various compositions were crosslinked into 200 μm-diameter gel cylinders via exposure to UV light (Supplementary Figures S2A and B). To determine *D*_g_, the substrates containing gel discs were incubated with tetramethylrhodamine (TRITC)-dextran MW 75 kDa, which served as a surrogate of the HGF. Supplementary Figure S2C shows a panel of images demonstrating the permeation of fluorescent dextran into hydrogel discs at various time points. This set of images highlights that hydrogels with 20 k PEG as the porogen proved to be the most permeable. Next, we plotted the ratio of fluorescence in the solution vs. fluorescence in the hydrogel as a function of time for several concentrations of 20 k PEG (Supplementary Figure S2D). We assumed that the fluorescence intensity is proportional to the concentrations of dextran, and we determined the diffusion coefficients of dextran through hydrogels by matching the experimental data (Supplementary Figure S2D) to the Fickian diffusion model with cylindrical coordinates using the COMSOL Multiphysics^39^. The effective diffusion coefficients for gels (*D*_g_) were determined to be 1.04×10^−7^, 0.20×10^−7^, and 0.04×10^−7^ cm^2^ s^−1^ for 5% PEGDA hydrogels with 20%, 10%, and 5% 20 k PEG porogen, respectively. A *D*_g_ value of 1.04×10^−7^ cm^2^ s^−1^ was the best match for the design criterion of *D*_g_/*D*_0_~10 presented in Supplementary Figure S1; therefore, the prepolymer solution composed of 5% PEGDA (MW 700 Da) and 20% PEG (MW 20 kDa) was chosen for hydrogel fabrication in this study.

Once the optimal hydrogel formulation was finalized, we integrated the hydrogel barriers into the microfluidic devices. A typical device (shown in Figure 1) contained two columns of PDMS posts at the interface between the cell culture and sensing channels. These posts served to confine the viscous prepolymer solution and to prevent it from spilling over into adjacent microfluidic channels. Figure 2a shows bright-field and fluorescence images of a microfluidic device with a hydrogel barrier that contained TRITC-labeled PEG for visualization purposes. As seen from these images, the hydrogel barrier had a width of ~80 μm and was confined to the space between PDMS posts. To characterize the diffusion of analyte through this hydrogel barrier, a solution of TRITC-dextran (75 kDa, 2.5 mg mL^−1^ in PBS) was introduced into the cell culture chamber. Figure 2b demonstrates the diffusion of TRITC-dextran into the gel and into the sensing chamber as a function of time. On the basis of the fluorescence intensity, ~20% of TRITC-dextran diffused across the hydrogel barrier after 90 min of incubation. This observation was in agreement with the modeling of gel diffusivity described in Supplementary Figure S2. The cost and complexity associated with the human recombinant HGF motivated us to use dextran of a similar molecular weight. It is worth noting that dextran is a commonly used surrogate molecule for protein diffusion studies^40,41^. We should also note that the focus of this optimization study is on a molecular mimic of the HGF—a relatively large protein. The diffusion of much smaller molecules associated with injury and inflammation, such as the TGF-β1 (MW 17 kDa), is expected to occur much more rapidly.

### Optimization of microbead assays for detecting the HGF and TGF-β1 in microfluidic devices

In this work, non-fluorescent capture microbeads (diameter=5.0–5.9 μm) and fluorescent detection beads (diameter=0.4–0.6 μm) were modified with anti-GF Abs and were used as sensing elements. As described in Figure 1a, the GFs became bound to either capture or detection microbeads, causing the two types of beads to aggregate, leading to an increase in fluorescence over time. The fluorescence signal from the 5 μm-diameter capture beads was monitored, and capture beads with a signal-to-background (S/B) ratio of 5 were deemed as positive. The process of setting the S/B threshold for the selection of positive beads was automated using the ImageJ software. Supplementary Figure S3 shows an example of two capture beads—one deemed as positive and the other deemed as negative—based on the fluorescence signal. It is of note that the specific and nonspecific aggregation of much smaller detection beads occurred in parallel with the binding of detection beads to capture beads. However, the aggregates from these alternative assembly processes were significantly smaller and were eliminated from consideration using the ImageJ software.

Another experiment was aimed at optimizing the ratio of capture beads to detection beads (C/D ratio). For instance, a low C/D ratio (that is, number of detection beads>>number of capture beads) may allow for a high signal but will also lead to a high background signal (that is, noise), whereas a higher C/D ratio may result in a low signal and a low background signal. Although one capture bead with a diameter of 5 μm may theoretically bind to ~400 detection beads (diameter=0.5 μm), a C/D ratio of 1/400 may not be the most effective due to the high background signal. Therefore, our objective was to maximize the signal-to-noise (S/N) ratio through the optimization of the C/D bead ratio. As seen in Supplementary Figure S4, a C/D ratio of 1/140 results in the highest S/N ratio (32.6); therefore, this bead formulation was used in subsequent experiments.

### Calibrating microbead biosensors for GF detection

The calibration curves for the HGF and TGF-β1 were constructed prior to cell experiments to characterize the relationship between microbead fluorescence and GF concentration. Sensing microbeads were challenged with different concentrations of recombinant HGF (0–40 pM) and TGF-β1 (0–300 pM) inside the microfluidic device at 37 °C. The choice of concentrations was based on ELISA measurements of the hepatocyte secretions in microfluidic devices^42^. In a typical calibration experiment, recombinant GFs were reconstituted in a culture medium (DMEM with 10% serum) to a desired concentration and then injected into a cell culture chamber. In parallel, microbeads were infused into the sensing chambers of a microfluidic device. In this way, the calibration experiment mimicked the real-life scenario of cell-secreted GFs diffusing across the hydrogel barrier into the sensing chamber. The change in fluorescence intensity of the capture microbeads was monitored for 90 min. This time period was chosen based on our observations (Supplementary Figure S5) that the signal became detectable after 45 min and stabilized after 90 min. The limit of detection was defined as signal exceeding noise (S/N) by a factor of 3 Ref.(43) and was determined to be ~6 and 21 pM for the HGF and TGF-β1, respectively. We should also note that the limits of detection achieved for the HGF and TGF-β using the microbead biosensors approached those achieved for commercial ELISAs (for example, those from R&D Systems).

### Hepatocyte cultures in microfluidic devices

**Figure 3:**
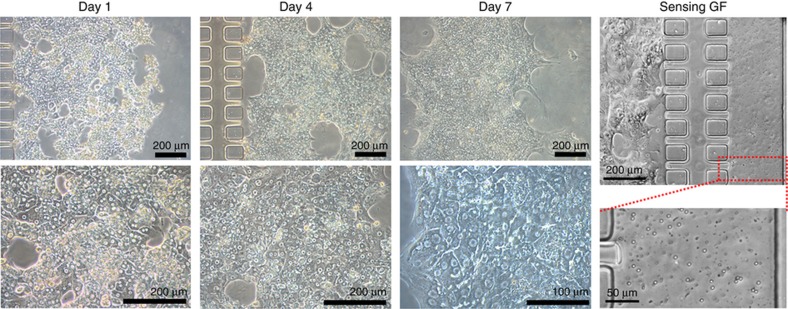
Hepatocyte cultures inside microfluidic devices. Cell cultures on days 1, 4, and 7. The sensing chambers are shown in the rightmost column. HGF, hepatocyte growth factor; TGF-β1, transforming growth factor-β1.

Primary hepatocytes were seeded into the collagen-coated microfluidic channels and were maintained for 7 days. As seen in Figure 3, these cells retained the epithelial phenotype, with prominent nuclei and distinct cell borders visible after 7 days of culture.

**Figure 4:**
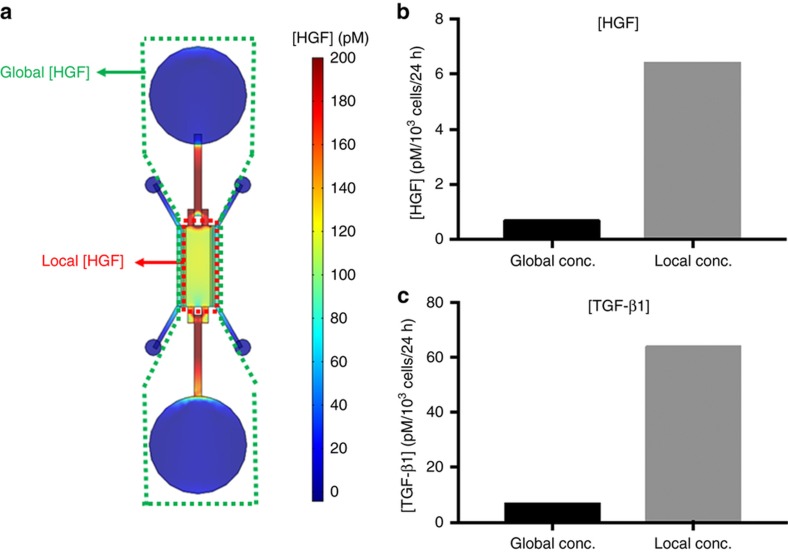
(**a**) Numerical simulation of HGF accumulation inside a microfluidic device at *t*=24 h based on the diffusion-reaction modeling. (**b** and **c**) Average global and local concentration (pM/10^3^ cells/24 h) of (**b**) HGF and (**c**) TGF-β1. HGF, hepatocyte growth factor; TGF-β1, transforming growth factor-β1.

Once cultivation of the hepatocytes in the microfluidic culture chambers was demonstrated, we sought to determine the levels of the HGF and TGF-β1 produced by the cells. We recently showed that these GFs are produced in larger amounts by hepatocytes inside microfluidic chambers compared to cells inside standard multiwell plates, and that endogenous GFs play an important role in shaping the hepatic phenotype in microfluidic channels^7^. The current paper is motivated by our desire to detect local concentrations of GFs close to cells vs. global concentrations that represent an average concentration of the GF in the whole microfluidic device. To highlight the difference between local and global concentrations, we modeled the secretion and transport of the HGF and TGF-β in our microfluidic device. The results of modeling for the HGF, shown in Figure 4a, demonstrate the establishment of HGF concentration gradients within the device, with ~7 pM present locally in the cell culture chamber and 0.67 pM being the global or average concentration inside the device. Similarly, the TGF-β1 concentration was approximated to be 60 and 8 pM in the cell culture chamber and in the whole microfluidic system, respectively. The volume of the device was dominated by the media reservoirs; therefore, the ELISA measurement yielded GF concentrations approaching that of the reservoir. This global measurement does not account for the morphogen gradients present in the device and underestimates the levels of these morphogens. Therefore, our strategy of placing sensing microbeads next to cells enabled sensing a higher local concentration of GFs.

**Figure 5:**
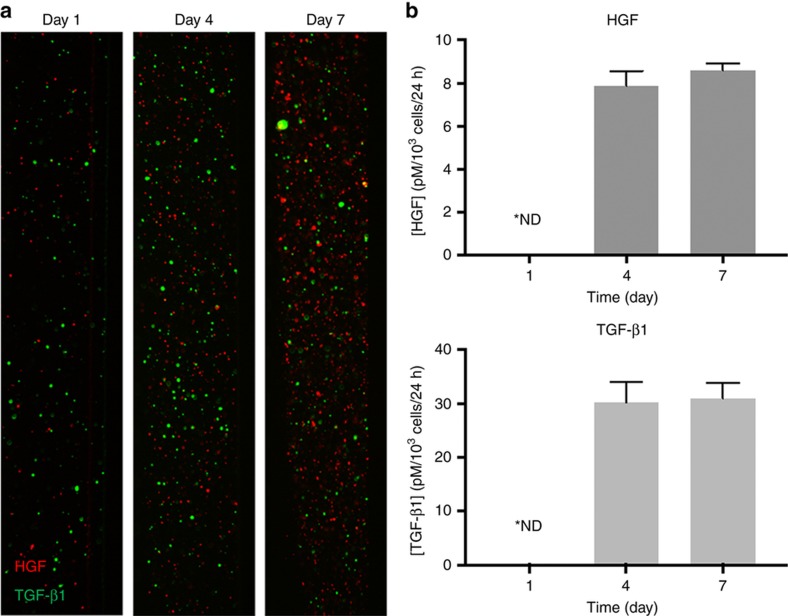
Simultaneous detection of the HGF and TGF-β1 secreted by hepatocytes cultured inside a microfluidic device. (**a**) Fluorescence images of sensing chambers detecting the HGF (red) and TGF-β1 (green) on days 1, 4, and 7. Scale bar=50 μm. (**b**) Average local concentrations of the HGF/TGF-β1 determined by comparing calibration curves to the fluorescence signals from cells (*ND, not detectable. Less than LOD). conc., concentration; HGF, hepatocyte growth factor; TGF-β1, transforming growth factor-β1. LOD, limit of detection.

**Figure 6:**
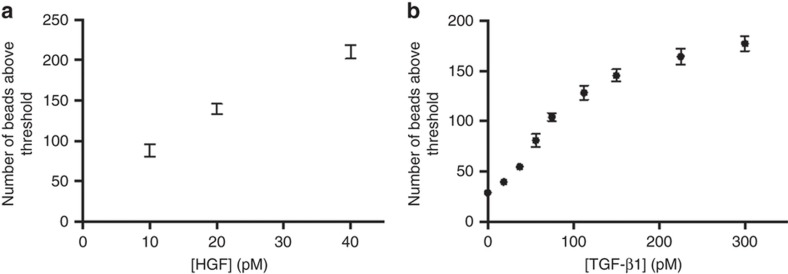
Calibrating microbead biosensors to correlate bead fluorescence with GF concentration. (**a**) HGF calibration curve and (**b**) TGF-β1 calibration curve, constructed by quantifying the number of fluorescent beads after 90 min of incubation with analytes (*n*=3). HGF, hepatocyte growth factor; TGF-β1, transforming growth factor-β1.

In the next set of experiments, microbeads functionalized with anti-HGF and anti-TGF-β1 were infused into a microfluidic device to monitor the secretion of these molecules by the hepatocytes. The microbeads for the HGF and TGF-β1 were indicated using Nile Red and Yellow fluorophores, respectively, for the simultaneous detection of these signaling molecules. In the process of bead introduction, the sensing channel was flushed out, removing the GFs that accumulated there during the time in the culture. We then monitored diffusion of GFs from the cell culture chambers into the sensing chambers for 90 min. These 90 min sensing sessions were repeated on days 1, 4, and 7. Figure 5a shows representative images of red and green fluorescent beads in the culture taken on different days. The numbers of fluorescent microbeads were converted into concentrations of GFs using the calibration curves described in Figure 6. These experiments revealed that the concentration of HGF ranged from 7 to 9 pM over the course of the experiments, whereas the concentration of TGF-β1 was ~30 pM (Figure 5b).

As a control experiment, we carried out a conventional ELISA for the TGF-β and HGF (Supplementary Figure S7). The media were collected from the inlet and outlet ports of a microfluidic cell culture device. The TGF-β levels were below the detectable limit on day 1, but then rose to 6 and 8 pM on days 4 and 7, respectively. These results of ELISA were ~4-fold lower than those of the microbead-based sensing of the local TGF-β concentration. These measurements supported the modeling prediction of several-fold differences between the average concentration of the TGF-β and the local concentration of this GF in the cell culture chamber described in Figure 4. We should note that the results of modeling predicted an eightfold difference, whereas the experimental findings showed a fourfold difference in concentrations. This discrepancy may be attributed to the oversimplifications and assumptions related to transport (particularly regarding the flow pattern) used by us for model construction.

We should also note that our results of modeling in Figure 4 predicted the global concentration of the HGF to be 0.7 pM, which is below the detection limit of a conventional ELISA (~2 pM for an R&D Systems assay). It was therefore unsurprising that the experimental ELISA measurements did not yield a detectable signal (data not shown).

One important consideration for us was the extent to which the process of sensing (capturing) GFs on microbeads affected the local GF concentrations in the vicinity of cells. A significant decrease in the local GF concentration is undesirable, as it may affect the autocrine signaling. A model was set up to compare the HGF concentration profiles in the presence or absence of sensing microbeads using the COMSOL Multiphysics software. This secretion-diffusion-reaction model incorporated many elements of our experiment, including the number of cells, GF secretion rates, dimensions of chambers, diffusivity of the hydrogel barrier, number of microbeads/Abs in the sensing channel, and rates of Ab-antigen binding. The results of this modeling are presented in Supplementary Figure S6. The black squares in Supplementary Figure S6 show the concentration profile of the HGF after 90 min inside the cell culture chamber in the absence of sensing microbeads while the red dots show HGF level after introduction of sensing microbeads into a microfluidic device. As seen from these data, the presence of microbeads had a minimal effect (~11% decrease) on the concentration of HGF inside the cell culture chamber. It should be noted that we have recently investigated how the depletion or dilution of cell-secreted signals affects the phenotype of hepatocytes^7^. On the basis of this recently published study, we conclude that an 11% depletion in the local concentration of the HGF or TGF-β should not have appreciable effects on cell function.

## Conclusions

This paper describes the development of a microfluidic device and microbead biosensors for the local monitoring of cell-secreted GFs. The need for such a microsystem is motivated by the increasing realization that cells function differently inside small volumes, and that endogenous signals play a greater role in microfluidic systems compared to standard large-volume cultures. Here we describe a microfluidic device that utilizes hydrogel barriers to separate cell culture chambers from sensing channels. The design of this device allows us to infuse sensing microbeads without perturbing neighboring cells for on-chip detection of local concentrations of important secreted factors. The composition of the hydrogel barrier is optimized to enhance the diffusion of GFs produced in the cell culture chamber. Furthermore, the microbead assay is optimized to enable the on-chip detection of HGF and TGF-β concentrations as low as 5.9 and 20.1 pM, respectively, in serum-containing cell culture media. Significant additional emphasis was placed on the calibration of the microbead sensors and on the construction of reaction-diffusion models to evaluate and optimize their performance. We believe that a combination of microfluidics and sensing microbeads provides a general framework for the local monitoring of cell-secreted signals. Unlike the immobilized or stationary biosensors that are used most commonly in conjunction with microfluidic devices, mobile microbead-based biosensors may be infused and flushed out at will and therefore do not suffer from saturation problems. The regeneration of such mobile biosensors is accomplished simply by infusing a new set of microbeads. Although sensing microbeads may be introduced directly into the microfluidic cell culture chamber, this strategy would be undesirable for cases of phagocytic cells. In addition, we are mindful of the fact that the local accumulation of secreted GFs and autocrine loops play a central role in defining the phenotype of cells inside microfluidic channels^42^. Introducing microbeads directly into the cell culture chamber would have meant the capture of a large fraction of secreted GFs and possible disruption of the autocrine signaling. The design of a microfluidic device with narrow, low-volume sensing channels adjacent to a much larger cell culture chamber alleviates this problem and allows us to use the high concentration of microbeads required for the sensitive detection of secreted factors while keeping GF consumption to a minimum (~11%). Furthermore, we demonstrate that microbead-based sensing may be multiplexed using different fluorescence labels. In upcoming studies, this cell culture and sensing microsystem will be used to monitor fibrogenic signals associated with liver injury. More broadly, this technology may be used to culture a variety of cell types while detecting multiple cell-secreted factors.
