# Supplementary material for: Detecting cell-secreted growth factors in microfluidic devices using bead-based biosensors
Source: Microsyst Nanoeng. 2017 Jul 3;3:17025. doi: 10.1038/micronano.2017.25 (PMC6023413; doi:10.1038/micronano.2017.25)
Supplement: Supplementary Figures [file micronano201725-s1.pdf]

Supplementary file

Detecting cell-secreted growth factors in microfluidic devices using bead-based biosensors

Kyung Jin Son[1], Pantea Gheibi[1], Gulnaz Stybayeva[1,2], Ali Rahimian[1,2] and Alexander Revzin[1,2]

Microsystems & Nanoengineering (2017) **3,** 17025; doi:10.1038/micronano.2017.25; Published online: 17 July 2017

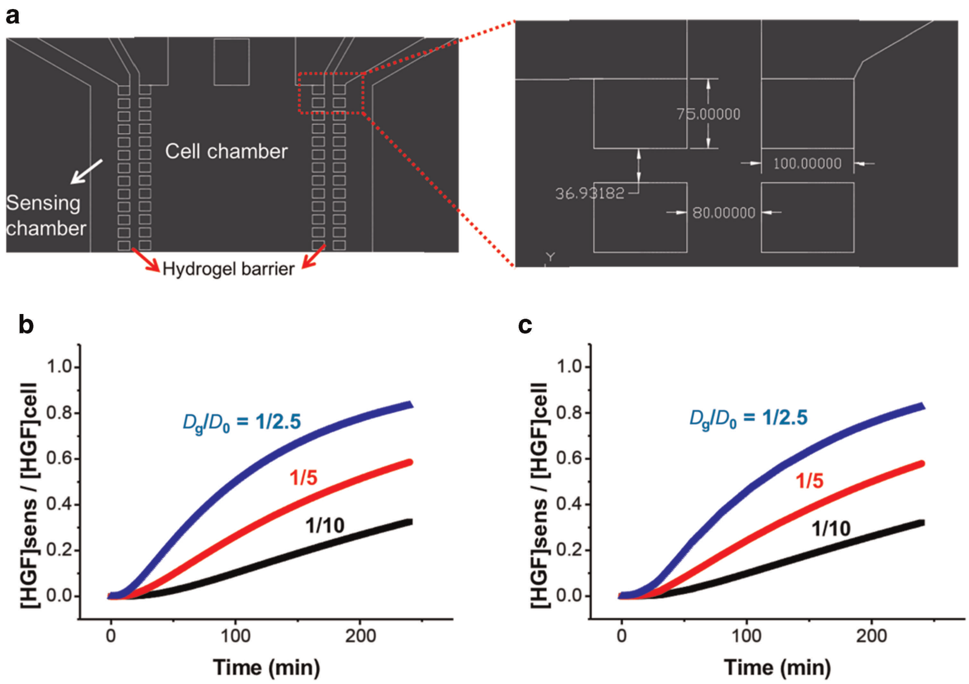

Figure S1 Design of microfluidic devices for cell culture and *in-situ* detection of growth factors released from cultured cells. (**a**) Design of the device. (**b** and **c**) Simulation data showing HGF concentrations in cell/sensing chambers with (**b**) 10 pM of recombinant HGF and (**c**) cells in the cell chamber, with varying ratio of HGF diffusivity in hydrogel, D_g, and HGF diffusivity in solution, D_0.

[1]Department of Biomedical Engineering, University of California, Davis, California 95616, USA and [2]Department of Physiology and Biomedical Engineering, Mayo Clinic, Rochester, Minnesota 55905, USA
Correspondence: Alexander Revzin (revzin.alexander@mayo.edu)

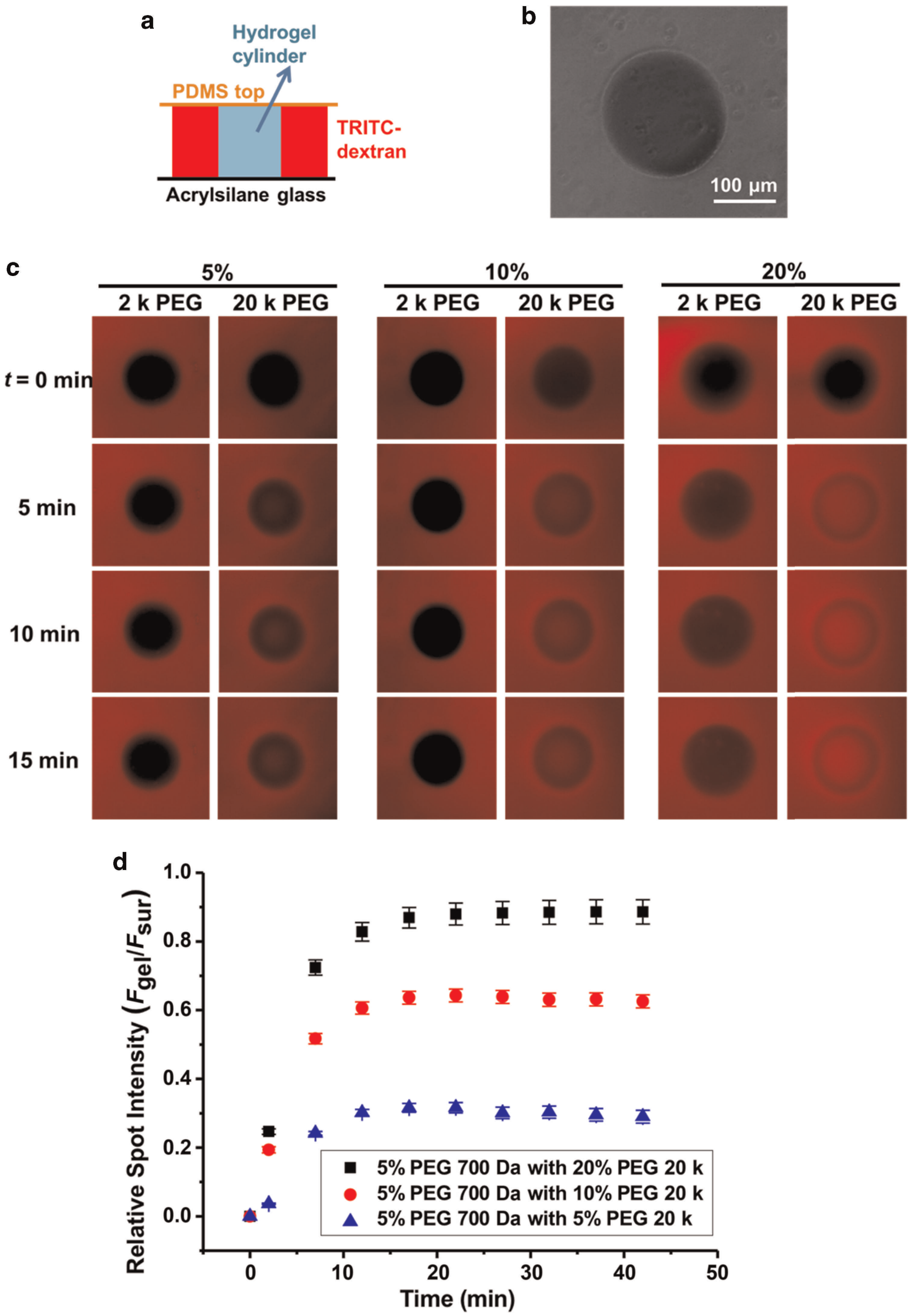

Figure S2 Optimization of hydrogels for rapid transports of growth factors from cell chamber to sensing chambers. (**a**) Hydrogel cylinders trapped inside microfluidic devices and surrounded by TRITC-dextran (75 kDa). (**b**) Bright-field image of hydrogel cylinder (5% PEG 700 Da with 20% PEG 20 k) inside microfluidic devices. (**c** and **d**) Fluorescence changes in hydrogel cylinders after incubation with TRITC-dextran for 15 min.

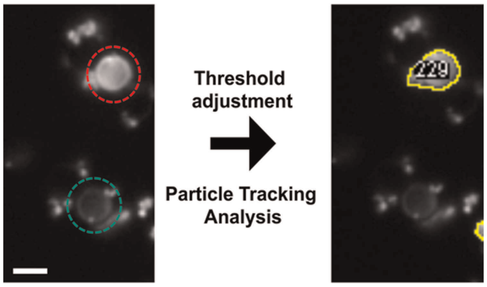

Figure S3 Analyzing fluorescence signal as the number of beads above threshold (≥5×(noise i.e., background signal)). The microbead exceeding threshold (red) is tracked and counted as signal while the microbead below threshold (green) is neglected. Scale bar: 5 μm.

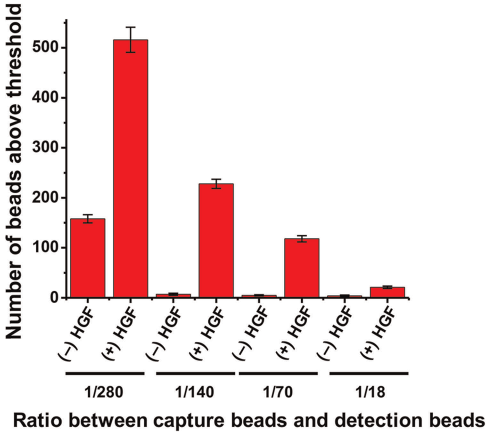

Figure S4 Optimization of the ratio between capture beads and detection beads for high signal with low background signal.

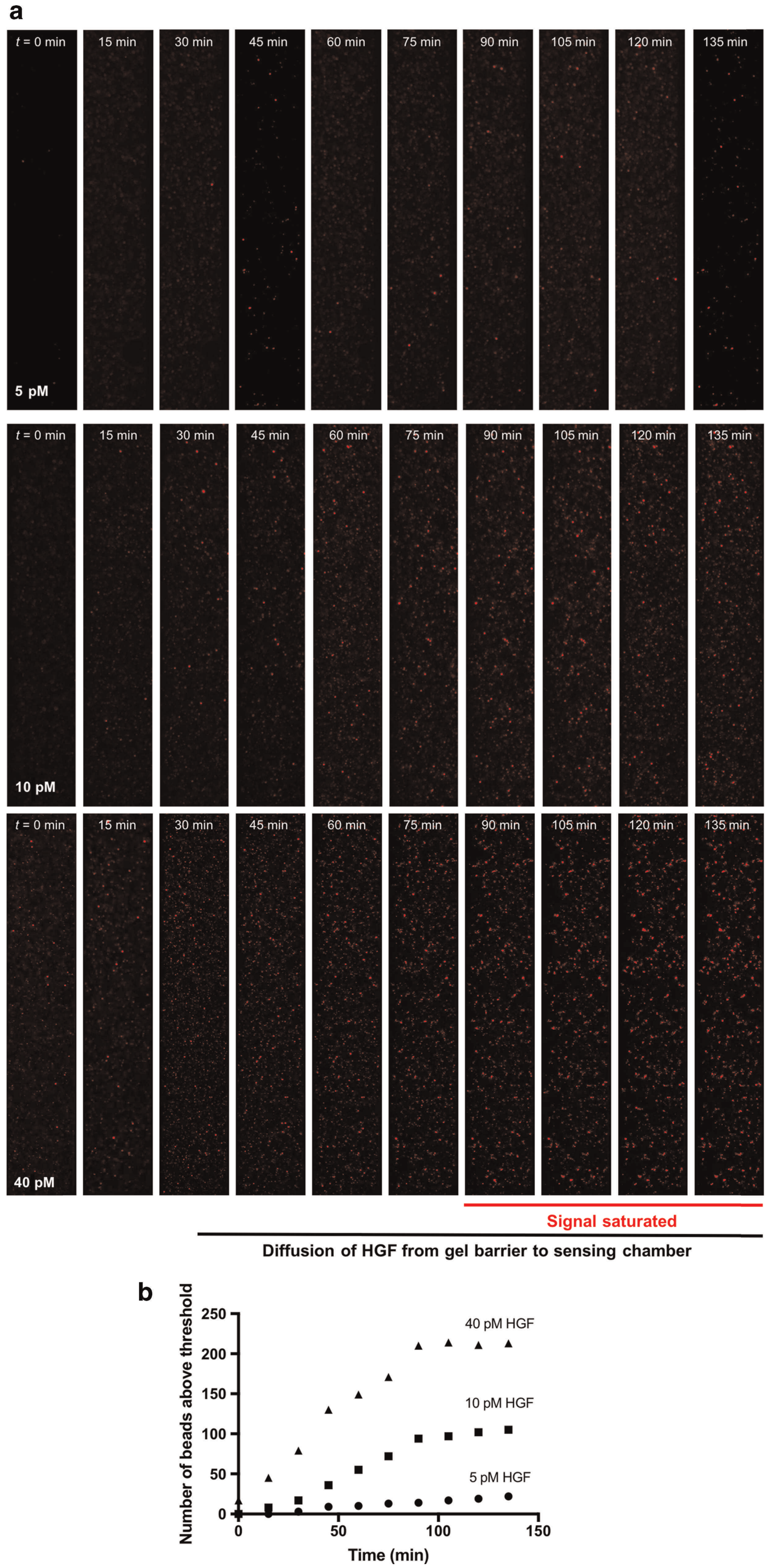

Figure S5 (**a**) Time-lapse fluorescence images of sensing beads inside sensing chamber in microfluidic devices. Recombinant HGF (conc. = 5, 10, 40 pM) was injected into cell chamber while beads solution was infused into sensing chamber. Fluorescence increases on beads were monitored for 135 min and fluorescence reached the point of saturation at $t = 90$ min. Scale bar: 100 μm. (**b**) Temporal response of a microbead assay challenged with varying concentrations of HGF.

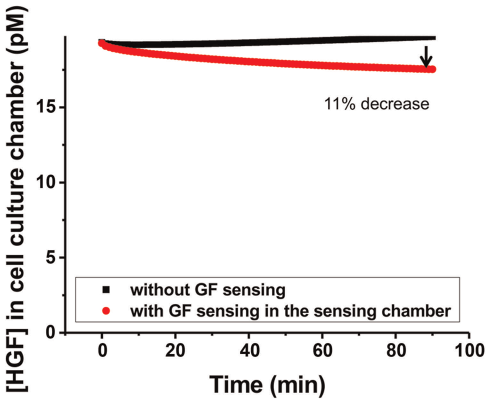

Figure S6 Concentration profile of HGF inside the cell culture chamber without GF sensing (black) and with GF sensing in the sensing chamber (red). Initial HGF concentration ($t = 0$ min) is the simulated concentration of HGF secreted from 2.1×10^3 hepatocytes with a secretion rate of 0.54 ± 0.02 pg/10^3 cells/24 h for 24 h.

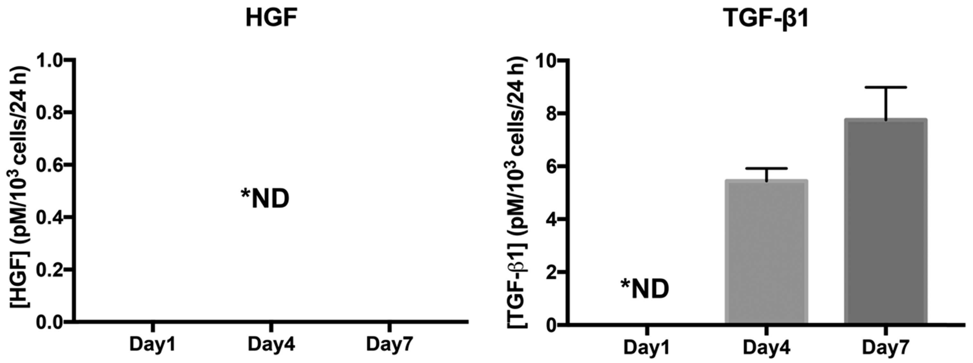

Figure S7 Global concentrations of cell-secreted HGF and TGF-β1 determined by ELISA. (*ND: Not detectable. Less than LOD).
